# Statistical evaluation of testing conditions on the saturated hydraulic conductivity of Brazilian lateritic soils using artificial intelligence approaches

**DOI:** 10.1038/s41598-022-24779-1

**Published:** 2022-11-27

**Authors:** Weber Anselmo dos Ramos Souza, Sávio Aparecido dos Santos Pereira, Thiago Augusto Mendes, Rafaella Fonseca Costa, Gilson de Farias Neves Gitirana Junior, Juan Félix Rodríguez Rebolledo

**Affiliations:** 1 Institut de Recherche en Mines et en Environnement, Université du Québec, Abitibi-Témiscamingue, Rouyn-Noranda, Québec Canada; 2 Federal Institute of Education, Science and Technology of Goias (IFG), Aparecida de Goiânia, Brazil; 3 School of Civil and Environmental Engineering, Federal University of Goias (UFG), Goiânia, Brazil; 4 Department of Civil, Construction, and Environmental Engineering, North Caroline State University, Raleigh, North Caroline USA; 5 Technology College, University of Brasilia (UnB), Brasília, Brazil

**Keywords:** Civil engineering, Engineering

## Abstract

The saturated hydraulic conductivity, *k*_*sat*_, is a crucial variable to describe the hydromechanical behavior of soils. The value of *k*_*sat*_ of lateritic soils that are typically found in tropical regions is highly affected by the soil’s structure, void ratio, and fine particle aggregation. As a result, the determination of *k*_*sat*_ in the field or in the laboratory is complex and involves greater variability, depending on the type of test and on the spatial location of sampling. This paper presents a study of *k*_*sat*_ values of lateritic soils, analyzing them using Statistic, Multilayer Perceptron Artificial Neural Networks (ANN) and Decision Trees (CHAID). This study aims to support decision-making regarding the type of test and depth chosen for sampling in laterite soils and understanding the factors influencing the permeability of such soils. An extensive literature review on the *k*_*sat*_ values of lateritic soils was performed, providing data for the establishment of a database comprise of 722 registries. According to agronomic and geotechnical soil classifications, the Brazilian lateritic soils presents a “moderate” hydraulic conductivity. A significant variation of permeability values along the depth was identified, particularly for depths between 0.1 and 0.2 m. Regarding the importance of testing variables, the ANN indicated a high dependency on the type of test. The decision tree divided field test and laboratory test automatically, inferring the relevance of the type of test to the determination of *k*_*sat*_.

## Introduction

The hydraulic conductivity of the soil (*k*) is defined, by Darcy's law, as the relationship between the percolation rate of water volume per unit of total area and the hydraulic head gradient. When *k* reaches its maximum value, it is called saturated hydraulic conductivity of the soil (*k*_*sat*_), a property that is dependent on the soil particle size distribution, particle morphology, pore continuity, particle orientation, volume of pores, among other factors^1–3^. The testing methodology for determining *k*_*sat*_, whether in-situ or laboratory, also influences its value.

The dependence of *k*_*sat*_ on these several factors turns this into a complex parameter with significant variability, reaching variations of over 200%^3–6^. In this context, understanding how *k*_*sat*_ is affected by soil characteristics, the type of test performed and the sample depth becomes essential for determining this parameter^7–9^.

In lateritic soils, *k*_*sat*_ becomes particularly affected by the soil structure. Moreover, this soil, typical of tropical regions from Africa, South America, and Southeast Asia^10,11^, are commonly formed from the weathering of rocks subjected to the high temperatures and humid climates typical of these regions^10,12,13^, and are characterized by often having a high void ratio, high clay content, and significant presence of iron and aluminum oxides and hydroxides, resulting in the aggregation of these fine particles^10,14^. Particle aggregation form macro and micropores in the soil, resulting in a bimodal pore-size distribution. This structure particularity of the soil pores offers preferential paths for water percolation, which can generate large variations in the values of *k*_*sat*_ when comparing different samples obtained from the same location. As a result, understanding the permeability phenomena in this soil type becomes challenging^13^.

The physical determination of *k*_*sat*_ may employ field or laboratory tests. In field tests, the soil does not undergo significant deformations in its structure, allowing a better understanding of the permeability phenomenon. However, it is difficult to control the boundary conditions of field tests. The main field tests used for determining *k*_*sat*_ are: Guelph permeater, slug test, pump test and concentric ring or double ring infiltrometer. The main laboratory tests available are: permeameters with constant and falling head, and triaxial tests.

Field and laboratory tests are costly and require considerable times^15^. For his reason, indirect estimation methods have been developed, such as theoretical equations^3,16^, pedotransfer functions^15,17,18^, and machine learning methods^19–21^. Machine learning methods, which can have either a regression or classification character, can be an essential tool for assessing the sensitivity of variables influewnced by complex relationships, as is the case for *k*_*sat*_.

This paper evaluates of the main factor influencing *k*_*sat*_ values of Brazilian lateritic soils using statistics, multilayer perceptron artificial neural networks (ANN), and a CHAID-type decision tree. The analyses presented herein contribute to the understanding of hydraulic conductivity in Brazilian lateritic soils aind in the establishemtn of adequate methodologies for the determination of representative *k*_*sat*_ values, according to the sample depth and test type.

## Materials and methods

### Data collection

First of all, a wide literature review was carried out on scientific articles that deal with the determination of the saturated hydraulic conductivity (*k*_*sat*_) of Brazilian lateritic soils, especially from the Midwest region. Test results on soils with the presence of roots were disregarded. The data collected included various types of equipment, testing methodologies, and variable soil sample depth. The following databases were used: (a) Scopus, (b) Web of Science, (c) ASCE and (d) Google Scholar, emphasizing the latter due to the ease, accessibility, and practicality in searching and obtaining journals, dissertations, and theses.

Search strings were established to identify publications presenting the measurement of the hydraulic conductivity of lateritic soils, considering various testing methodologies, equipment, and depths. Initially, the geographic location was not specified since lateritic soils are present in several Brazilian locations. There was no limitation on the publication period for the scientific articles researched. Combination of words for publications written in Portuguese were: ("permeabilidade" AND "ensaio" AND "laterítico") OR ("condutividade hidráulica" AND "ensaio" AND "laterítico") OR ("condutividade hidráulica saturada" AND "ensaio" AND "laterítico"). The corresponding combinations of words for publications written in English were ("permeability" AND "test" AND "lateritic") OR ("hydraulic conductivity" AND "test" AND "lateritic") OR ("saturated hydraulic conductivity" AND "test" AND "lateritic").

**Figure 1:**
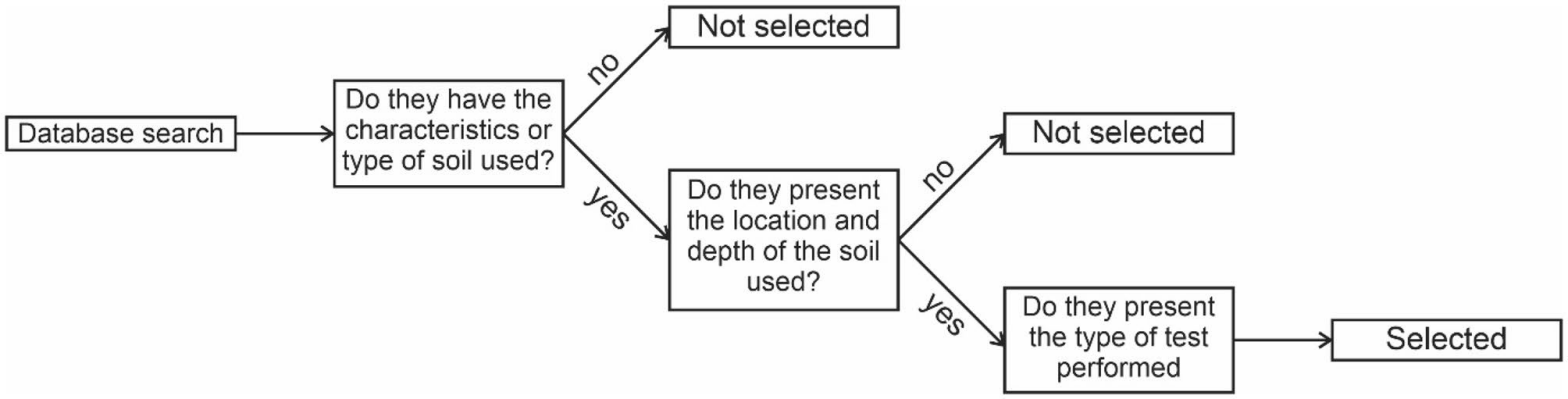
Criteria for selecting publications.

Altogether, 6414 scientific articles were found: 60 from Scopus, 10 from Web of Science, 154 from ASCE, and 6190 from Google Scholar. Because of the large number of articles found, these were screened based on the rejection criteria shown in the flowchart presented in Fig. 1.

Using the collection of *k*_*sat*_ data obtained in the literature review, a database was built to support statistical and computational analyses, aiming to understand better the influence of the type of test, method or equipment and depth of sample. Machine learning methods, including Artificial Neural Networks (ANN) and decision trees, were used as described later.

### Materials

**Figure 2:**
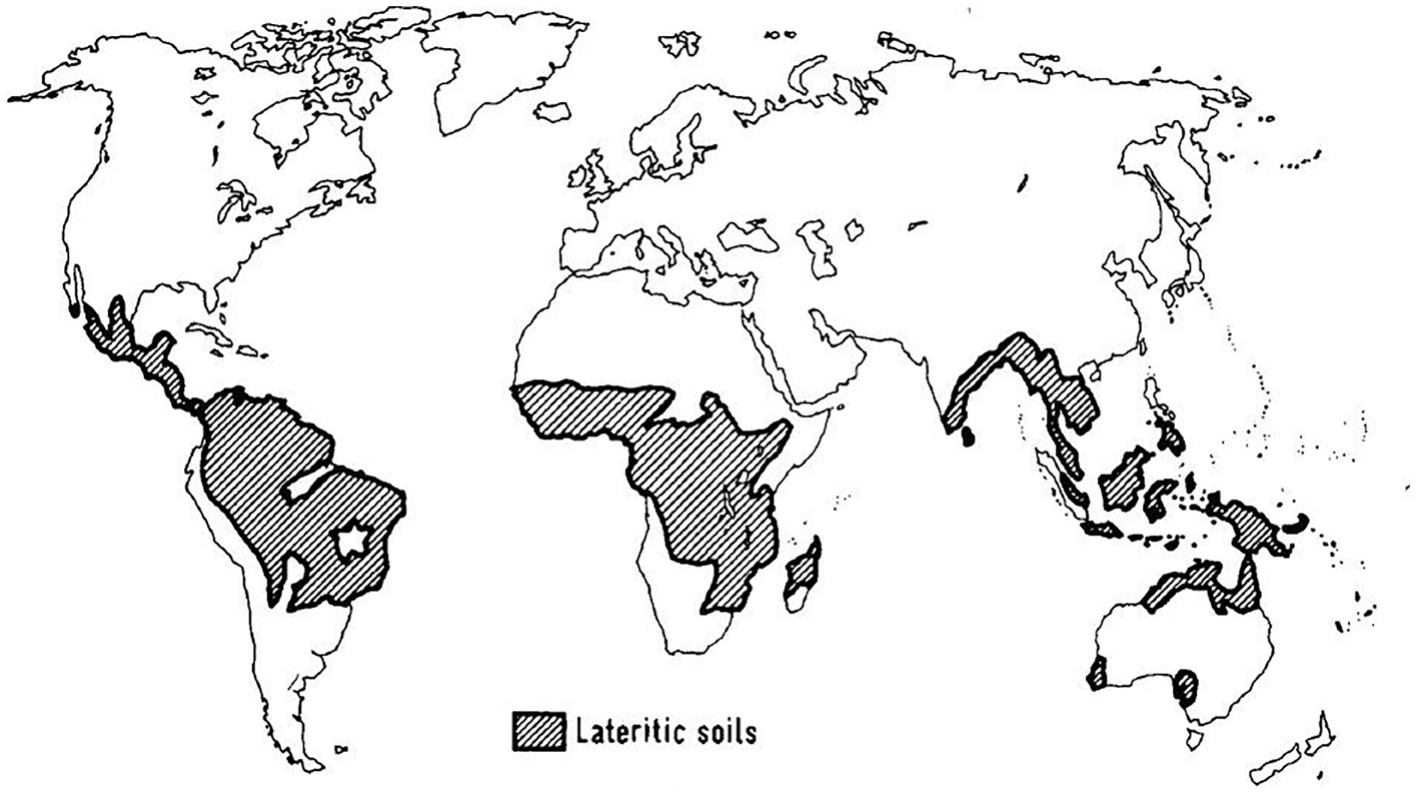
Regions with the occurrence of lateritic soils (Charman^23^).

Data used in this paper comprise exclusively lateritic soils. According to Fortes and Merighi^22^, lateritic soils’ colors are yellow and red because of aluminum hydroxides and ferric hydrates. The Unified Soil Classification System (USCS) is generally considered not suitable for tropical soils. In fact, lateritic materials are better characterized and classified using the MCT (Miniature, Compacted, Tropical) classification system. In terms of location, lateritic soils are typically found in the regions indicated by the Charman^23^ map (Fig. 2).

The magnitude of the *k*_*sat*_ of tropical soils can be classified according to the flow rate ranges established by Ferreira (1999) apud Freire et al*.*^24^, and presented in Table 1. It could be noted that the values shown in Table 1 are for agronomic studies, and were initially presented in meters day^−1^, being converted to meters seconds^−1^ in this paper. A permeability classification for use in civil works was not found in the literature. However, a classification of permeability values by type of soil was published by Das and Sobhan^25^, as shown in Table 2.

**Table 1:** Saturated hydraulic conductivity classes established by Ferreira (1999) apud Freire et al.^24^.

Classes	*k*_*sat*_ (m s^−1^)
Very fast	> 6.94 × 10^–5^
Fast	3.47 × 10^–5^–6.94 × 10^–5^
Moderately fast	1.74 × 10^–5^–3.47 × 10^–5^
Moderately	5.56 × 10^–6^–1.74 × 10^–5^
Moderately slow	1.39 × 10^–6^–5.56 × 10^–6^
Slow	3.47 × 10^–7^–1.39 × 10^–6^
Very slow	< 3.47 × 10^–7^

**Table 2:** Saturated hydraulic conductivity classes presented by Das and Sobhan^25^.

Classes	*k*_*sat*_ (m s^−1^)
Clear gravel	1 × 10^0^–1 × 10^–2^
Coarse sand	1 × 10^–2^–1 × 10^–4^
Fine sand	1 × 10^–4^–1 × 10^–5^
Silty clay	1 × 10^–5^–1 × 10^–7^
Clay	< 1 × 10^–8^

Regarding the types of tests on laterite soils, the two main laboratory apparatuses used to determine *k*_*sat*_ are the constant load and the falling head permeameters. These tests are standardized in Brazil through technical standards NBR 13,292^26^ and NBR 14,545^27^, respectively. Undisturbed and remolded specimens can be used in these tests, with the constant head test being commonly adopted for granular soils, while the falling head test is aimed at clay soils with relatively low permeability.

The main approached for determining *k*_*sat*_ in the field are the Guelph test, the slug test, the pump test, and the concentric rings test. The Guelph apparatus is commonly used, being practical, easy to perform, having a low cost, and offering a quick means of determination of *k*_*sat*_. The Guelph apparatus is generally used for small depths, up to 75 cm^28,29^. The slug test is a field test that consists of inserting a cylinder into the soil and monitoring the flow of water.

The pump test is a more complex procedure that uses a suction pump to assess variations in water flow in the soil. The concentric ring method consists of placing two rings of different diameters on the soil surface. Water is added to the two circles, where an initial reading of the water height is performed and readings are taken at predetermined time intervals, evaluating the height variation in the inner circle to calculate the hydraulic conductivity. This method does not have many difficulties in its execution and results in more homogeneous values. Unfortunatelly, the concentric ring method offers overestimated values of *k*_*sat*_, due to the imposed hydraulic head^30^**.**

### Description of the ANN used

The software used for building the ANN was IBM SPSS Statistics^31^, adopting a multilayer perceptron network^32–34^. The multilayer perceptron network is a commonly adopted supervised learning method among the various ANN methodologies developed. The approach requires the availability of training data, used to adapt the model. This network is commonly used to evaluate databases, images, and other types of data^35–38^, and consists of a set of layers subdivided into: input, hidden, and output layers. The input layer consists of the data region used for training, while the output layer returns the desired parameter. The hidden layer is an intermediate layer and aims to connect the input values with the output values. The connections between each layer are made through weights, which are values assigned initially at random, and later adjusted by the ANN during training. Weights are assigned from the input layer to the hidden layer and from the hidden layer to the output layer.

The values that feed the hidden layer come from the scalar product between the assigned weights and the input layer values, being applied through a mathematical function called activation function, which has the purpose of linearizing the data^39^. The most used activation functions are the ReLU and the hyperbolic tangent^34^. The final value that comes out of the hidden layer is the scalar product applied to the activation function arriving at the output layer^39^. The resulting value that leaves the output layer is the value predicted by the ANN^39^.

To minimize the errors computed during training, a backpropagation process was used: the application of the descending gradient, an interactive method of non-linear optimization^33,34^. The descending gradient method requires two critical parameters: the learning rate and the momentum. The learning rate determines the learning speed of the algorithm. In general, values between 0 and 1 are adopted. Momentum is a parameter that gives the network stability, allowing for rapid convergence. Like the learning rate, its values vary between 0 and 1.

Another parameter determined during training is the Bias, a unitary component used to compensate for random weight assignments^34^. Its incorporation into an ANN is essential, as it allows the translation of the scalar product between the components of a layer and their respective weights, preventing the ANN from assigning greater weight to a component of a specific layer due to the lack of freedom of movement of the scalar product.

**Figure 3:**
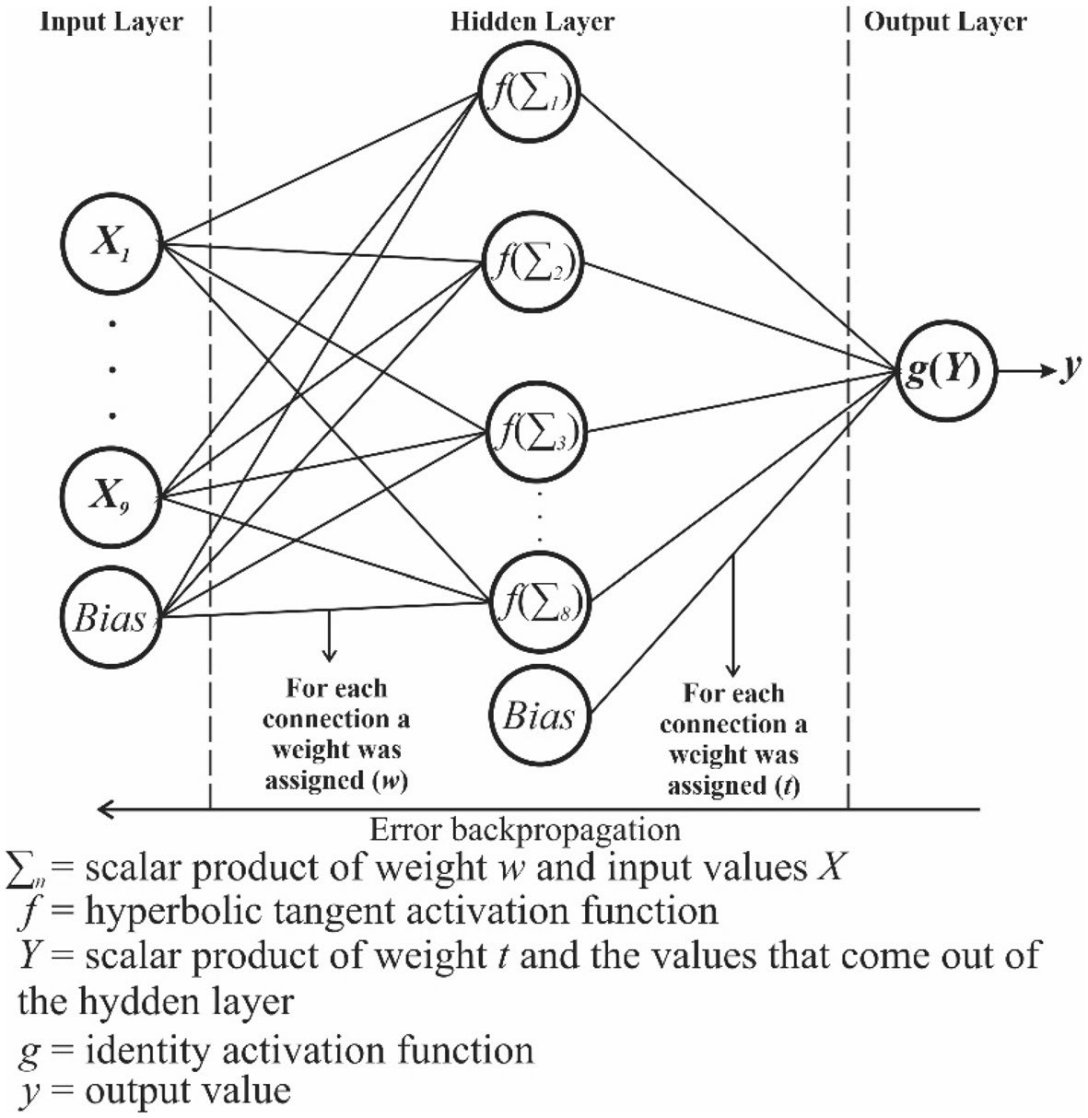
Diagram of the ANN adopted.

Figure 3 outlines the ANN model used, based on the concepts, parameters and methodology described herein. As activation functions, hyperbolic tangent and identity were used for the input and the output, respectively. In the modeling of the ANN, a single hidden layer was used. The ANN method with a multilayer perceptron network used in this paper offers also a quantitative assessment of the importance of the variables used in the determination of *k*_*sat*_, through the assigned weights.

A fraction of 69.3% of the data was used for network training, 20.1% for testing, and 10.6% for model validation (holdout). In the validation, cross-validation was adopted, and the training was carried out in batches. Cross-validation is characterized by dividing the data into a specific integer value *n*, selecting for each *n* a percentage of data for training and another for testing. The process is repeated for each division *n* and the model with the best fit is adopted. The initial learning rate was 0.4 and the moment was 0.9.

### Chis-square automatic interaction detector (CHAID) decision tree

According to Biggs et al.^40^, decision tree and classification techniques are powerful tools used for dividing analysis data into homogeneous groups. One of these methods is the automatic interaction using chi-square (CHAID), implemented through an algorithm developed by Kass^41^ and improved by Biggs et al.^40^. The population subdivision criteria must meet the selected statistical significance, maximum depth of the tree and minimum number of cases (parent node and child node). If the criteria not reached, the values are not divided concerning the studied variable^42^.

According to Kass^41^, CHAID comprises the following steps: (a) best partition for each predictor; (b) the predictors are compared and the best one is chosen; (c) the data are subdivided according to the predictors and the division criteria; (d) each subgroup is independently analyzed to produce other subdivisions.

The advantages of CHAID are its straightforward interpretation and reading, little computational time and the possibility of having multiple divisions in a node^42,43^. Regarding the disadvantages, the method requires a large amount of data to achieve adequate results^44^. This method was used herein to evaluate the influence of the different variables involved (types of tests, method or equipment and sample depth) in the determination of *k*_*sat*_, proposing a class of the importance of these variables through the generated model (CHAID), is also used for validating the results of ANN modeling.

## Results and discussions

### Overview of selected publications

**Figure 4:**
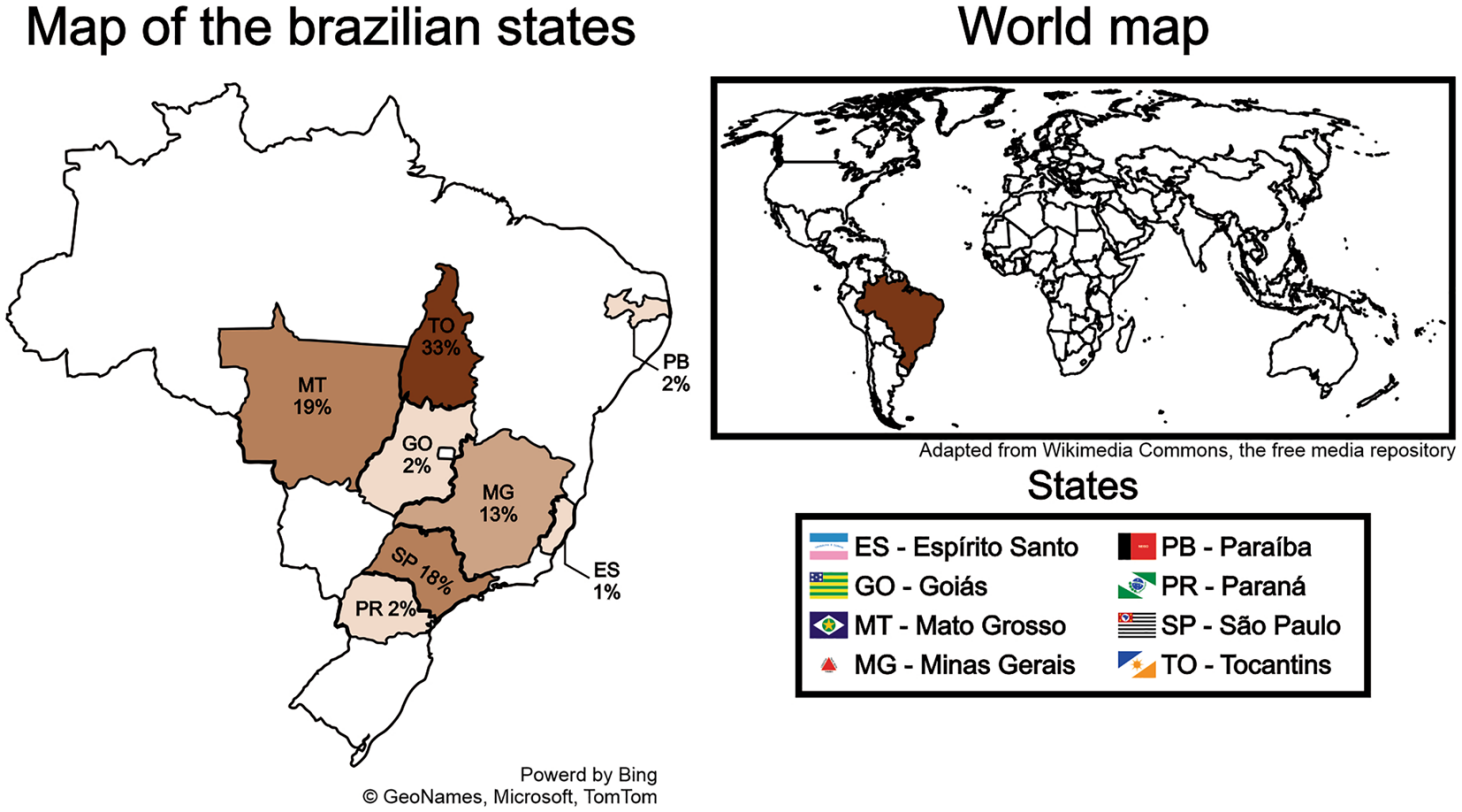
Geographic distribution of selected *k*_*sat*_ data.

A total of 6414 scientific papers, theses and dissertations were found, but only 18 were selected, 7 of which were doctoral theses^45–51^, 6 master's dissertations^52–57^, 1 monography^58^ and only 4 scientific papers^14,59–61^. In addition to the documents searched in the databases, the *k*_*sat*_ data presented in the Brazilian Agricultural Research Corporation (EMBRAPA) bulletin, Filizola et al.^9^, was also included. The geographic distribution of the *k*_*sat*_ data selected is shown in Fig. 4.

**Figure 5:**
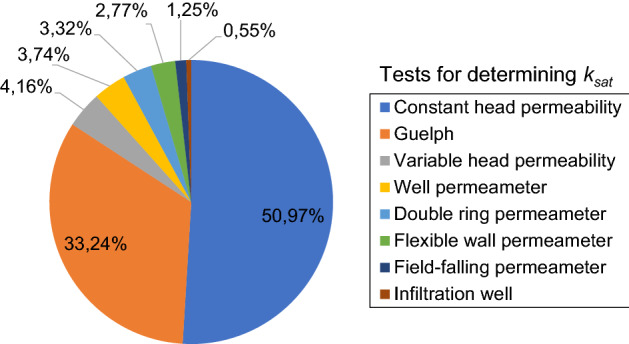
Distribution of the methods used in the collected data.

The selected publications presented results obtained using the constant head, the falling head, the Guelph, the double ring, the flexible wall, and the field-falling permeameters. Some studies also presented data obtained from infiltration well, and well pumping tests. The depths of the tests or samples varied from 0.00 to 3.00 m. Figure 5 shows the distribution of *k*_*sat*_ registries by each method. The most common laboratory permeability test was the constant head, and the most common field test was the Guelph test. According to Elmashad and Ata^62^, the most common field test is the ring infiltrometer, but not the Guelph test.

### Analysis of collected data

The main statistical parameters describing the collected soil permeability dataset are presented in Tables 3, with *k*_*sat*_ values in their original form and in the natural logarithmic scale. Table 3 shows higher coefficient of variation (COV) values, while logarithmic ones show a significant reduction. This reduction is associated with the scaling effect of the logarithm operator.

**Table 3:** Data statistics for *k*_*sat*_ values and absolute values of *ln*(*k*_*sat*_).

Group	Min	Max	Median	Mean	Std. Dev	COV %	N
**Original scale**							
Constant head	4.00E−09	1.22E−03	1.13E−05	1.56E−05	6.32E−05	404.8%	368
Falling head	6.56E−09	7.40E−05	2.08E−06	6.90E−06	1.47E−05	212.6%	30
Guelph	2.11E−08	1.50E−03	8.58E−06	7.17E−05	1.77E−04	246.9%	240
Double ring permeameter	4.17E−07	8.30E−05	8.89E−06	1.69E−05	2.25E−05	133.3%	24
Flexible wall permeameter	2.70E−09	1.42E−05	6.08E−06	6.59E−06	5.94E−06	90.0%	20
Field-falling permeameter	2.64E−07	1.33E−05	4.73E−06	5.79E−06	5.19E−06	89.6%	9
Infiltration well	1.60E−04	1.52E−03	7.77E−04	8.09E−04	7.38E−04	91.3%	4
Well permeameter	1.00E−06	1.00E−04	1.00E−05	3.09E−05	3.85E−05	124.7%	27
**Natural logarithmic scale (absolute values)**							
Constant head	6.71	19.34	11.39	11.53	0.92	8.0%	368
Falling head	9.51	18.84	13.14	13.29	1.98	14.9%	30
Guelph	6.50	17.67	11.67	11.59	2.19	18.9%	240
Double ring permeameter	9.40	14.69	11.63	11.91	1.56	13.1%	24
Flexible wall permeameter	11.16	19.73	12.01	13.06	2.35	18.0%	20
Field-falling permeameter	11.23	15.15	12.26	12.73	1.48	11.6%	9
Infiltration well	6.49	8.74	7.60	7.61	1.23	16.2%	4
Well permeameter	9.21	13.82	11.51	11.11	1.23	11.1%	27

*COV* Coefficient of variation, *N* number of samples.

**Figure 6:**
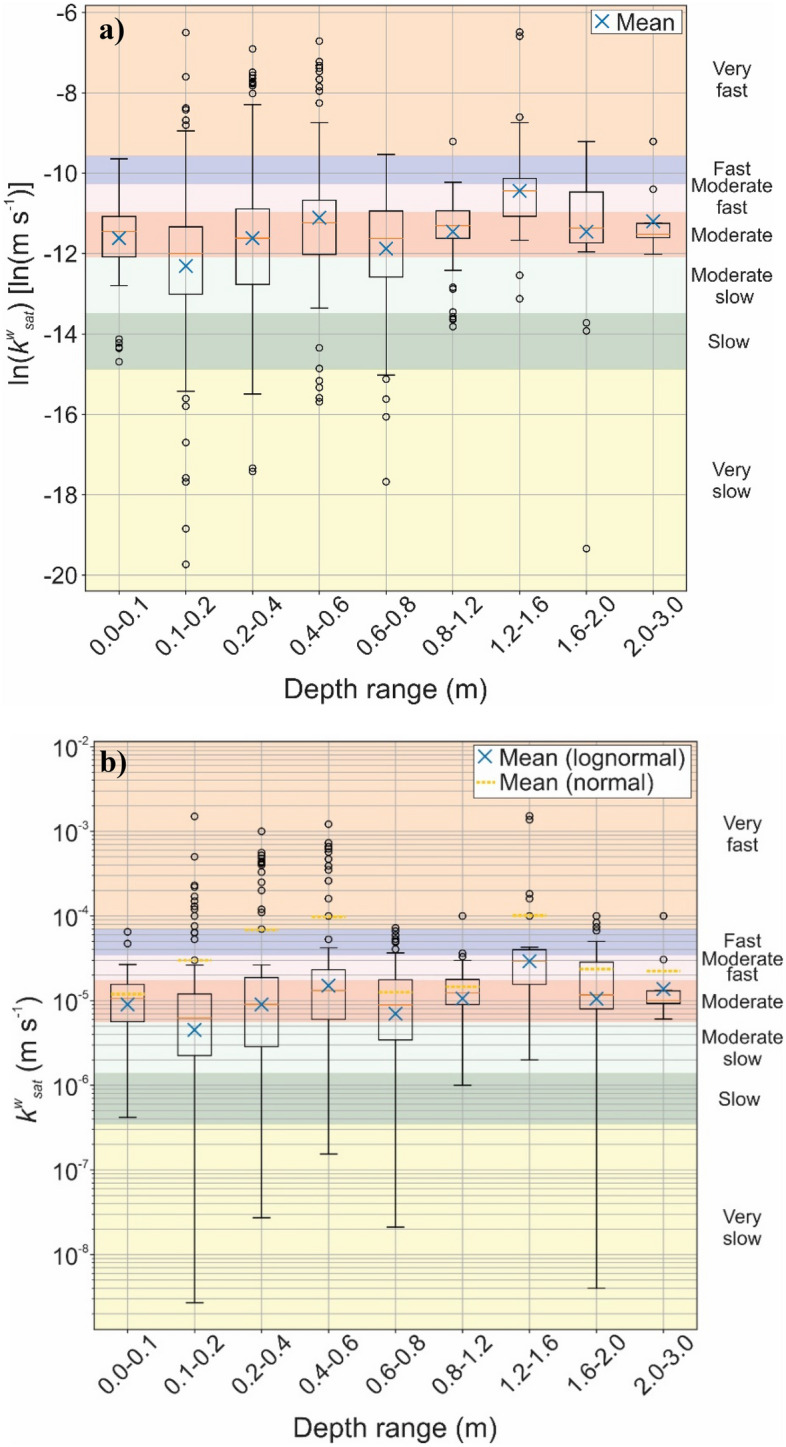
Distribution of saturated hydraulic conductivity of lateritic soils by depth range, classified according to Table 1: (**a**) ln *k*_*sat*_; (**b**) *k*_*sat*_.

**Figure 7:**
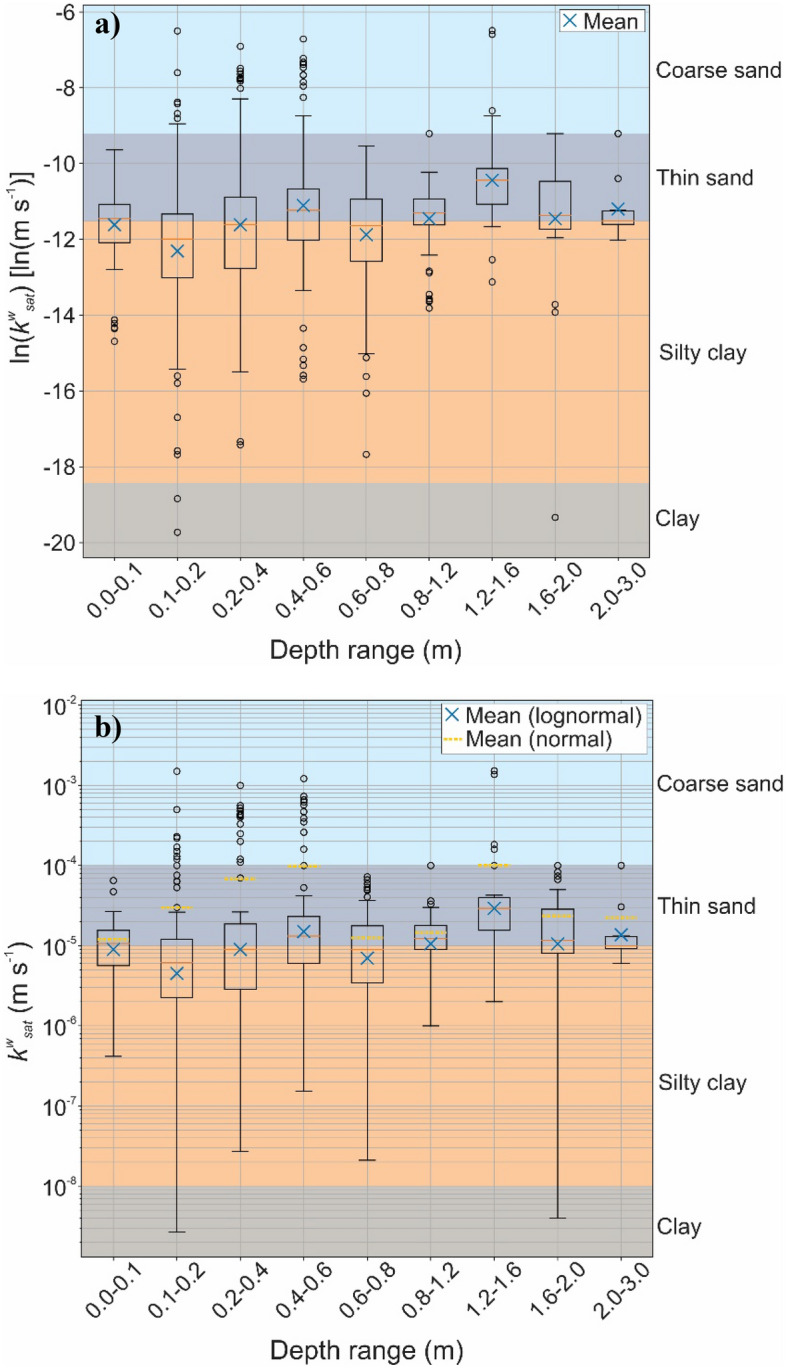
Distribution of the saturated hydraulic conductivity of lateritic soils by depth range, classified according to Table 2: (**a**) ln *k*_*sat*_; (**b**) *k*_*sat*_.

The *k*_*sat*_ values of the Brazilian lateritic soils surveyed were classified according to the flow rate ranges shown in Tables 1 and 2. The corresponding ranges, in terms of infiltration speed are shown along with the box diagrams presented in Figs. 6 and 7. The box plot limits are presented for *p* = 0.05.

The values of the natural logarithm of *k*_*sat*_ (Fig. 6a) were plotted in the box diagrams, considering that these values follow the lognormal distribution, as suggested by Gitirana and Fredlund^63^, and the original *k*_*sat*_ values (Fig. 6b). It is important to note that the original *k*_*sat*_ values have an asymmetric distribution. The use of permeability values in their original form, as shown in Fig. 6b, leads to the identification of outliers only above the superior range limit. These evidences indicate that the statistical interpretation of the natural logarithm of *k*_*sat*_ may provide more intuitive and useful information.

It can be inferred from Fig. 6a that the values between the first quartile and the third quartile, comprising 50% of the dataset, in addition to the median and average data, were in the “moderately slow”, “moderate” and “moderate fast” classification classes. According to the criteria proposed by Ferreira (1999) apud Freire et al*.*^24^, this indicates that the infiltration capacity of the studied lateritic soils is high. Although there is a wide variety of textural characteristics among Brazilian oxisols, previous studies have not shown a clear relationship between these characteristics and hydraulic properties^59^. The dataset presents numerous outliers with respect to the depth data, both above and below the range limits, reflecting its wide sample variability. This fact can be explained by the different types of tests used to obtain the *k*_*sat*_ values.

Figure 6a shows that most of the *k*_*sat*_ data surveyed fall within the range classified as “very fast”, mainly for low depths, up to 60 cm, then moving to the “fast” class for depths between 0.6 and 1.20 m, and increasing again to the “very fast” class for greater depths of the soil profile i.e., higher than 1.20 m).

In Fig. 7, the *k*_*sat*_ values are presented along with depth ranges and separated according to the ranges shown in Table 2. In Fig. 7a, the permeability values are presented in the natural logarithm, while in Fig. 7b the values are presented on their original scale. Again, it is possible to notice that, in Fig. 7b, the diagram is skewed, since the hydraulic conductivity data do not follow the normal distribution and are asymmetric^63^. A significant portion of the values is found in the range of clay with silt and fine sand, showing the moderate infiltration capacity of lateritic soils presented in Fig. 6a.

**Figure 8:**
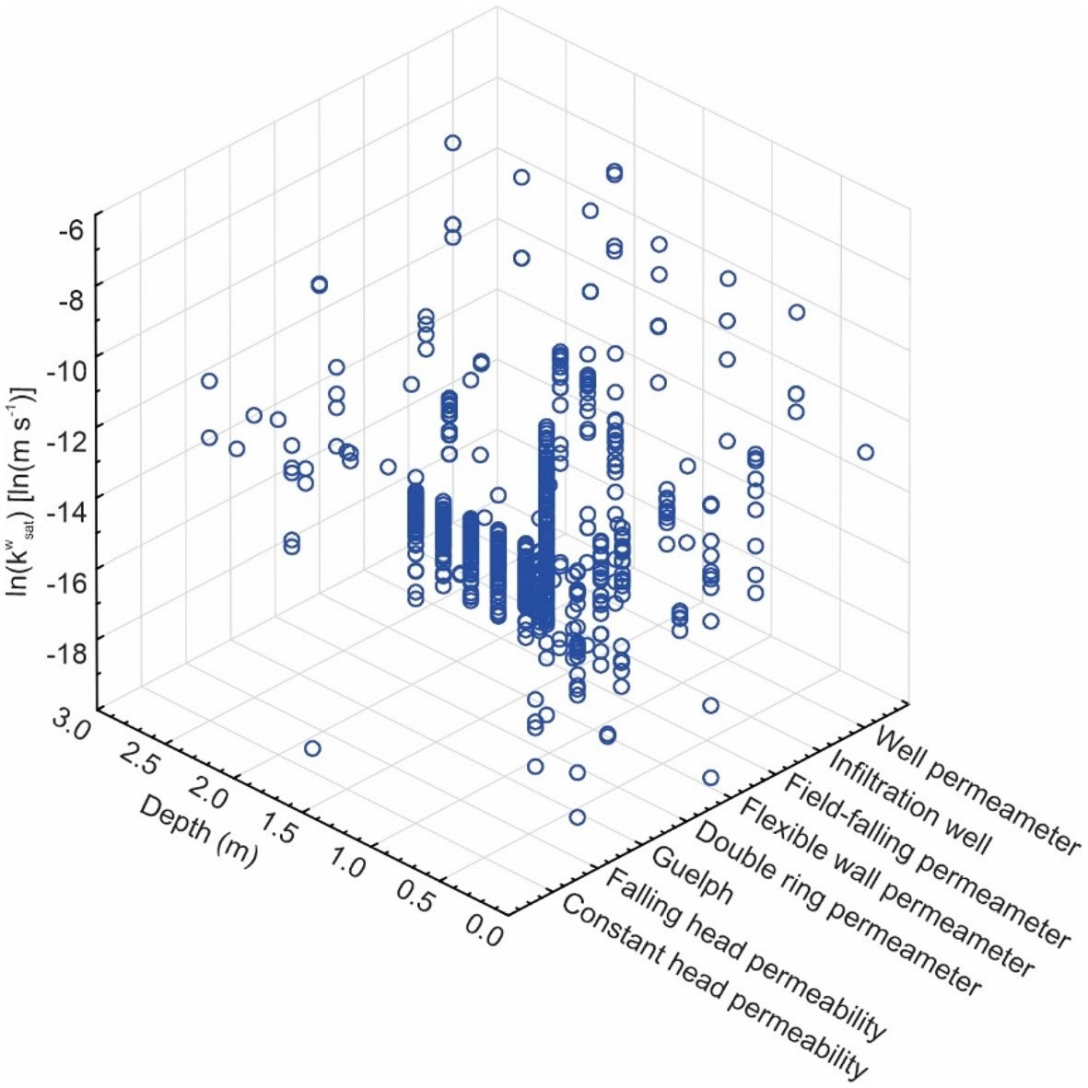
Distribution of the saturated hydraulic conductivity of the lateritic soil by depth range and type of test by different authors, considering all the researched data.

To assess the influence that the type of equipment, instrument or test method may have on the determination of *k*_*sat*_, three approaches were adopted: (a) 3D graphing; (b) Artificial Neural Networks (ANN) and (c) CHAID Decision Tree. Figure 8 shows all the *k*_*sat*_ data, with an average *k*_*sat*_ equal to 4.5 × 10^–6^ m s^−1^. The dataset is separated according to the type of permeameter (constant head, falling head and Guelph) and infiltrometer. This average value is similar to that obtained using triaxial permeameter tests, as presented by Mendes^64^, Vaz^65^, and Araújo^54,66^, all for lateritic soil from the city of Goiânia, state of Goiás, Brazil.

It could be noticed that the average *k*_*sat*_ values are overestimated when combining all depths (Fig. 8). This is, once again, a results of the skewed distribution, as presented in Fig. 6a.

**Figure 9:**
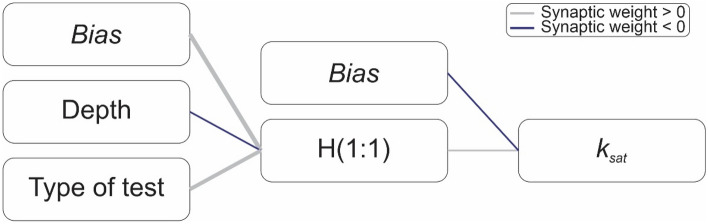
ANN approach for the evaluation of the hydraulic conductivity data of the lateritic soils.

Figure 9 presents the procedure for assessing the influence of testing conditions (i.e., test type and testing depth) using the ANN. In Fig. 9, each box represents the layer variables that return the value of the next layer. The thickness of the connections between each box represents the modulus of the weight assigned to that value in determining the value of the next layer. The greater the thickness of the connection, the greater the value of the modulus of the weight of that element. In addition, the weight module signals are represented in gray for positive values and blue for negative values. The weight signals are interpreted as being directly proportional in determining the value of the next layer, when positive, and inversely proportional when negative.

A sensitivity analysis was made based on the training and testing samples to determine each predictor's importance in the neural network. These values represent the relative importance for the main predictor. The testing method had a 82.2% relative influence, whereas the testing depth has a 17.8% relative influence. Figure 9 indicates that the type of equipment, instrument and test performed has a stronger connection with the hidden layer of the ANN and, consequently, more significant importance in the determination of *k*_*sat*_ than the sample depth.

**Figure 10:**
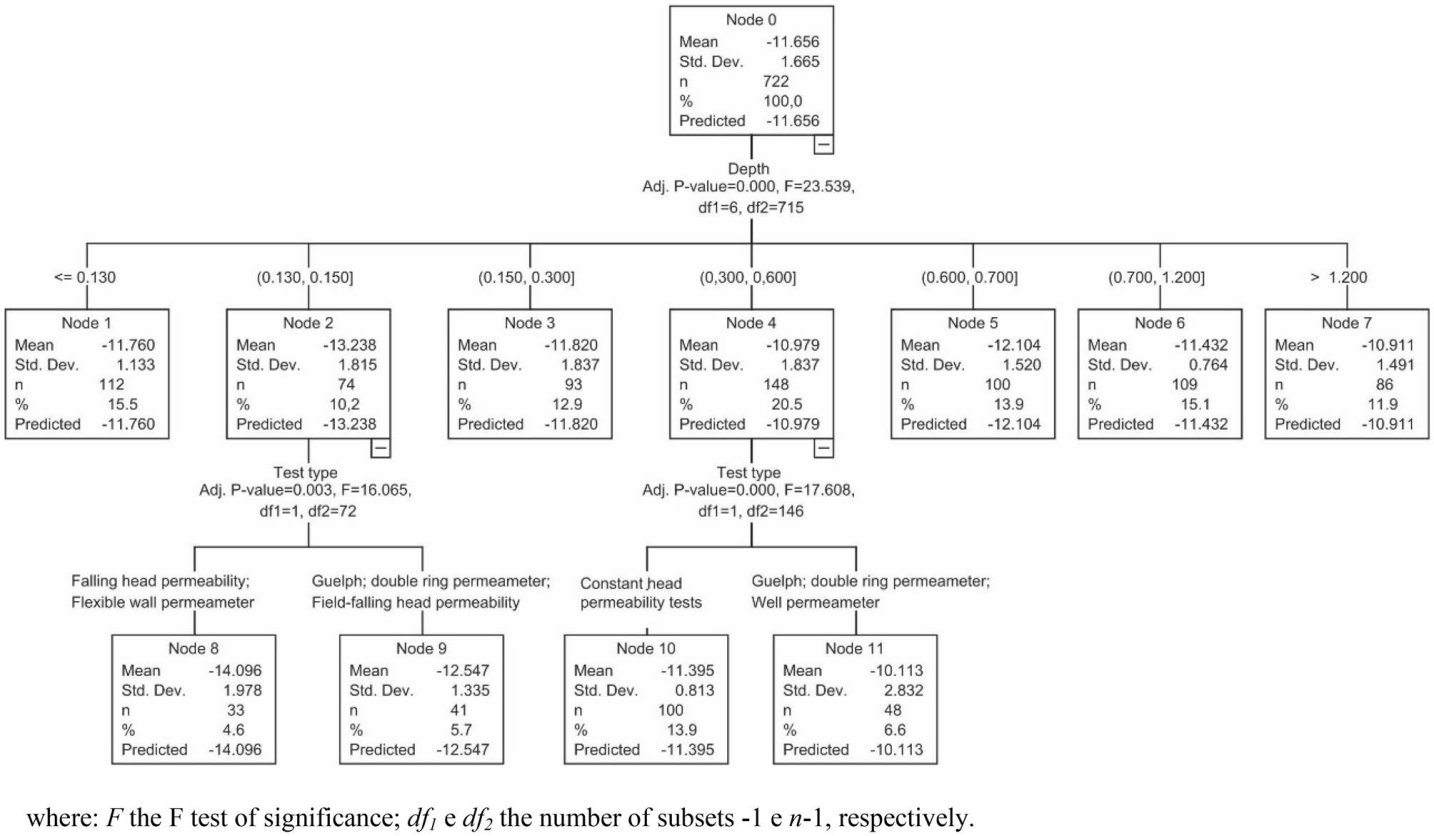
CHAID decision tree for hydraulic conductivity values of Brazilian lateritic soils surveyed.

Comparing the results obtained using the ANN (Fig. 9), a decision tree of the CHAID type (Fig. 10) was developed using a division criterion node relative with a minimum of 60 tests, and child node with a minimum of 30 trials. The CHAID decision tree has three classification levels: the upper level (Node 0), determined by the *k*_*sat*_ values; the intermediate level (Nodes 1–7), showing the depths of sampling/testing and the last level (Nodes 8–11), with the types of test used for determining *k*_*sat*_.

The levels of the decision tree indicate the hierarchy or degrees of relative importance of the parameters and variables evaluated by the algorithm, the first level being the *k*_*sat*_ values. This level was subdivided into seven branches or nodes (Nodes 1–7), with the statistical parameters of each node and its significance being indicated in each node box (Fig. 10).

For Nodes 2 and 4, that is, the most superficial depths and the most suitable for hydrological and agronomic studies (0.13–0.15 and 0.3–0.6 m), there was a division of the *k*_*sat*_ values as the representativeness of the testing method (Nodes 8–11). Thus, the algorithm classified for each node the type of test (field and laboratory) most likely recommended for the determination of *k*_*sat*_, according to the testing depths, indicating the values that are closer to the mean and standard deviation.

Figure 10 indicates that the permeability values obtained from laboratory and field tests have characteristics in common between them. This finding may allow the optimization of testing programs and reduce costs and result in testing time savings due to the similarity between the methods. Furthermore, it was possible to observe that the *k*_*sat*_ values obtained from laboratory tests are lower than those obtained from field tests. Some authors have reported this finding previously, such as Gribb et al.^67^, Elfaki^68^, and Elhakim^69^. For the other nodes (other depths evaluated), there was no significant difference in *k*_*sat*_ obtained in the field or in the laboratory, as perceived by the CHAID decision tree generator algorithm.

In general, the results demonstrate a significant relevance of the type of test and sample depth to the measured *k*_*sat*_ values of Brazilian lateritic soils. The statistical assessment of the testing approach is essential for the analysis of the results obtained in testing programs. A high variability in the *k*_*sat*_ values was observed both considering the type of test and the sample depth. COV values of 247% were observed for the Guelph test and 405% for the constant head. Along the depth a variation between 10^–2^ and 10^–9^ m s^−1^ was observed for the *k*_*sat*_ values. Considering the average values of *k*_*sat*_, it was possible to verify that, despite its variability, the average values were similar when comparing determinations using the same type of test and the same depth. Moreover, the constant head permeameter determinations and testings on samples obtained between 0.7 and 1.2 m in depth showed the lowest standard deviations. As a result, these testings specifications (i.e., constant head on specimens between 0.7 and 1.2 m) should result in more consistent permeability data.

## Conclusions

According to the results and analyses presented herein, the the following conclusions may be drawn from this study:

1. Most saturated hydraulic conductivity data published for Brazilian lateritic soils corresponds to laboratory tests using constant and falling head permeameters (around 65%) and Guelph field tests (around 35%), These tests where carried out for depths between 10 cm and 3 m. The obtained values of *k*_*sat*_ are usually classified as “moderate” for agronomic purposes and correspond to the behavior of sands, from a geotechnical engineering perspective.
2. The artificial neural networks (ANN) and the CHAID decision tree proved to be efficient tools to support the selection of testing methodology, the depth of execution of the tests for the determination of *k*_*sat*_, and to raise awareness regarding difficulties in interpreting results obtained for Brazilian lateritic soils. For instance, the depths of soil profiles between 0.13 to 0.6 m showed the highest standard deviation values. Therefore, it is necessary to provide carefull interpretations of testing results obtained from this depth range.
3. The ANN showed that the type of test has more influence on the value of *k*_*sat*_ than the sampling depth (about 4.6 times superior significance). It is important to note that the soil condition (e.g., landfills, cuts, pre-densification) may also play a role in the value of *k*_*sat*_, requiring further studies.
4. It could be noted that the CHAID decision tree indicated that it is possible to separated field test and laboratory test in the same data sample. In general, the results obtained with the CHAID decision tree indicated lower values of *k*_*sat*_ obtained by laboratory tests than those performed in the field. Moreover, the constant head permeability and the range of depth between 0.7 and 1.2 m. showed the lowest standard deviation values. Therefore, this type of test and this interval of depth was suggested for lateritic soils.

Finally, it is important to emphasize that the results presented herein do not replace traditional testing programs. These results may provide preliminary estimations of *k*_*sat*_. The reported statistical values may aid the design of testing programs, allowing a better understanding of variability of testing results.
